# Predictors of miscarriage in polycystic ovary syndrome patients with threatened abortion: development and validation of a nomogram model

**DOI:** 10.3389/fendo.2025.1689878

**Published:** 2026-01-09

**Authors:** Luhang Ma, Qingdiao Zhou

**Affiliations:** 1 Department of Gynecology, Wenzhou People's Hospital, Wenzhou, Zhejiang, China; 2 Department of Obstetrics, Wenzhou People’s Hospital, Wenzhou, Zhejiang, China

**Keywords:** polycystic ovary syndrome, threatened abortion, nomogram, prediction model, influencing factors

## Abstract

**Objective:**

To investigate the risk factors associated with failed pregnancy maintenance in patients with polycystic ovary syndrome (PCOS) presenting with threatened abortion.

**Methods:**

Based on clinical diagnostic outcomes, 150 PCOS patients with early threatened abortion (gestational age ≤12 weeks) were categorized into two groups: a successful pregnancy maintenance group (n=100) and a failed pregnancy maintenance group (n=50). Relevant clinical parameters were collected, and binary logistic regression analysis was performed to identify independent risk factors for failed pregnancy maintenance. A nomogram prediction model was constructed using R software (version 4.21). The discriminative ability of the nomogram was evaluated using receiver operating characteristic (ROC) curve analysis, and a calibration curve was generated to assess model performance. Decision curve analysis (DCA) was employed to determine clinical utility.

**Results:**

The nomogram prediction model identified the following independent risk factors for failed pregnancy maintenance in PCOS patients (P < 0.05): testosterone levels, fasting insulin, and fasting blood glucose. These factors were incorporated into the final nomogram. The area under the ROC curve (AUC) ranged from 0.635 to 0.955, indicating strong discriminative power. The calibration curve closely approximated the ideal curve, demonstrating excellent model fit. Furthermore, decision curve analysis revealed that the model’s clinical utility was superior to extreme scenarios, confirming its practical value.

**Conclusion:**

Three clinical variables were independently associated with failed pregnancy maintenance in PCOS patients with threatened abortion. The developed prediction model, based on these variables, exhibits high accuracy and clinical applicability, providing a reliable tool for risk stratification and clinical decision-making.

## Introduction

Polycystic ovary syndrome (PCOS) is a complex endocrine disorder characterized by heterogeneous clinical manifestations, including ovulatory dysfunction, polycystic ovarian morphology, and hyperandrogenism or insulin resistance. As a reproductive-endocrine-metabolic disorder affecting 5.6% of women of reproductive age in China (1, 2), PCOS has a global prevalence of 10–13% (2). A key pathogenic factor contributing to infertility in PCOS patients is the impaired developmental competence of oocytes, involving alterations in cytoplasmic maturation and molecular composition (3). Notably, PCOS represents the most prevalent cause of anovulatory infertility (4), accounting for 50–70% of ovulation-related infertility cases (5). Emerging epidemiological data indicate an escalating incidence with a trend toward younger onset (6, 7).

Clinically, PCOS manifests as menstrual irregularities and infertility. Even when conception occurs, luteal phase deficiency frequently predisposes to threatened abortion or pregnancy loss (8). Compromised endometrial receptivity and aberrant trophoblast invasion further exacerbate reproductive challenges, increasing risks of miscarriage and gestational complications (9, 10). Threatened abortion (TA), defined as vaginal bleeding with/without abdominal pain before 28 gestational weeks without cervical dilation or fetal expulsion (11), progresses to spontaneous abortion in 20–50% of PCOS cases – significantly exceeding rates in non-PCOS populations (12, 13). These patients also face elevated risks of gestational hypertension, preeclampsia, fetal growth restriction, and preterm delivery (14), imposing substantial physical and psychological burdens.

The pathophysiological basis for this increased miscarriage risk in PCOS is multifactorial. Hyperandrogenism stands as a cornerstone risk factor. Elevated testosterone levels have been consistently linked to miscarriage, potentially by impairing endometrial receptivity through defective decidualization and creating a suboptimal environment for embryo implantation and early development (8, 10). Concurrently, insulin resistance (IR) and compensatory hyperinsulinemia are present in a majority of PCOS patients (15). This metabolic milieu can disrupt the placental-maternal interface by promoting chronic inflammation, oxidative stress, and inadequate trophoblast invasion, ultimately leading to placental dysfunction and pregnancy loss (9, 16). Furthermore, a state of subclinical hypercoagulability is often observed in PCOS, driven by factors such as obesity and IR, which can elevate coagulation factors like Factor VIII and predispose to placental microthrombosis, thereby compromising blood flow and leading to miscarriage (17, 18).

Current management of PCOS-related TA remains challenging, with a lack of universally standardized protocols. Modern approaches primarily focus on progesterone supplementation for luteal phase support and, in cases with underlying insulin resistance, the use of metformin (19, 20). Concurrently, Traditional Chinese Medicine is also explored as a complementary approach with therapeutic potential (21). However, the persistence of risk factors such as hypercoagulability (17, 18, 22) and psychological stress (23, 24) often leads to therapeutic failures, underscoring the suboptimal efficacy of current strategies and a pressing need for better prognostic tools. A critical gap persists in personalized risk prediction; clinicians lack a robust method to integrate multifaceted risk factors into a quantifiable estimate of miscarriage risk for the individual patient presenting with TA. Therefore, the objective of this study was to develop and validate an integrated predictive model that synthesizes key risk factors from multiple domains. By employing a nomogram approach, we aim to provide a clinically applicable tool that enables early identification of PCOS patients with TA who are at the highest risk of miscarriage, thereby facilitating timely and targeted interventions to improve pregnancy outcomes.

## Material and methods

### Reporting guidelines

The development and validation of the prediction model in this study strictly followed the TRIPOD (Transparent Reporting of a multivariable prediction model for Individual Prognosis Or Diagnosis) Statement (25).

### Study design and participants

This retrospective cohort study was conducted at Wenzhou People’s Hospital from May 2020 to June 2023. The study followed a model development and validation design using data from the same cohort. A total of 150 PCOS patients diagnosed with threatened abortion were consecutively enrolled. Based on treatment outcomes, participants were divided into two groups: the successful pregnancy maintenance group (n=100) and the failed pregnancy maintenance group (n=50). This study was approved by the hospital ethics committee.

All enrolled patients diagnosed with threatened abortion received a standardized treatment regimen according to the hospital’s clinical guidelines, aimed at supporting early pregnancy. The core management included: 1) Luteal Phase Support: All patients received progesterone supplementation. The primary modality was oral dydrogesterone (Duphaston^®^) administered at a dose of 10 mg, three times daily. In cases of significant vaginal bleeding, micronized vaginal progesterone (Utrogestan^®^) at a dose of 200 mg, twice or three times daily, was used as an alternative or adjunctive therapy. 2) Lifestyle Advice: Patients were advised to maintain bed rest, avoid heavy physical exertion, and refrain from sexual intercourse during the episode of bleeding. 3) Adjunctive Therapy for PCOS: For patients with a pre-existing diagnosis of IR or those identified with significant IR during admission (HOMA-IR ≥2.77), metformin was continued or initiated at a dose of 500 mg, three times daily, following consultation with an endocrinologist.

The treatment was initiated upon diagnosis and continued until 12 weeks of gestation if the pregnancy remained viable, or until the occurrence of a miscarriage. This consistent protocol across the cohort ensures that the identified risk factors are independent predictors of outcome despite standard-of-care treatment. This retrospective study was conducted according to the TRIPOD-AI reporting guideline. No pre-registered protocol was prepared, but all analytic steps were predefined before data extraction and analysis. This study did not involve patients or the public in the design, conduct, or reporting of the research due to its retrospective nature.

**Figure 1:**
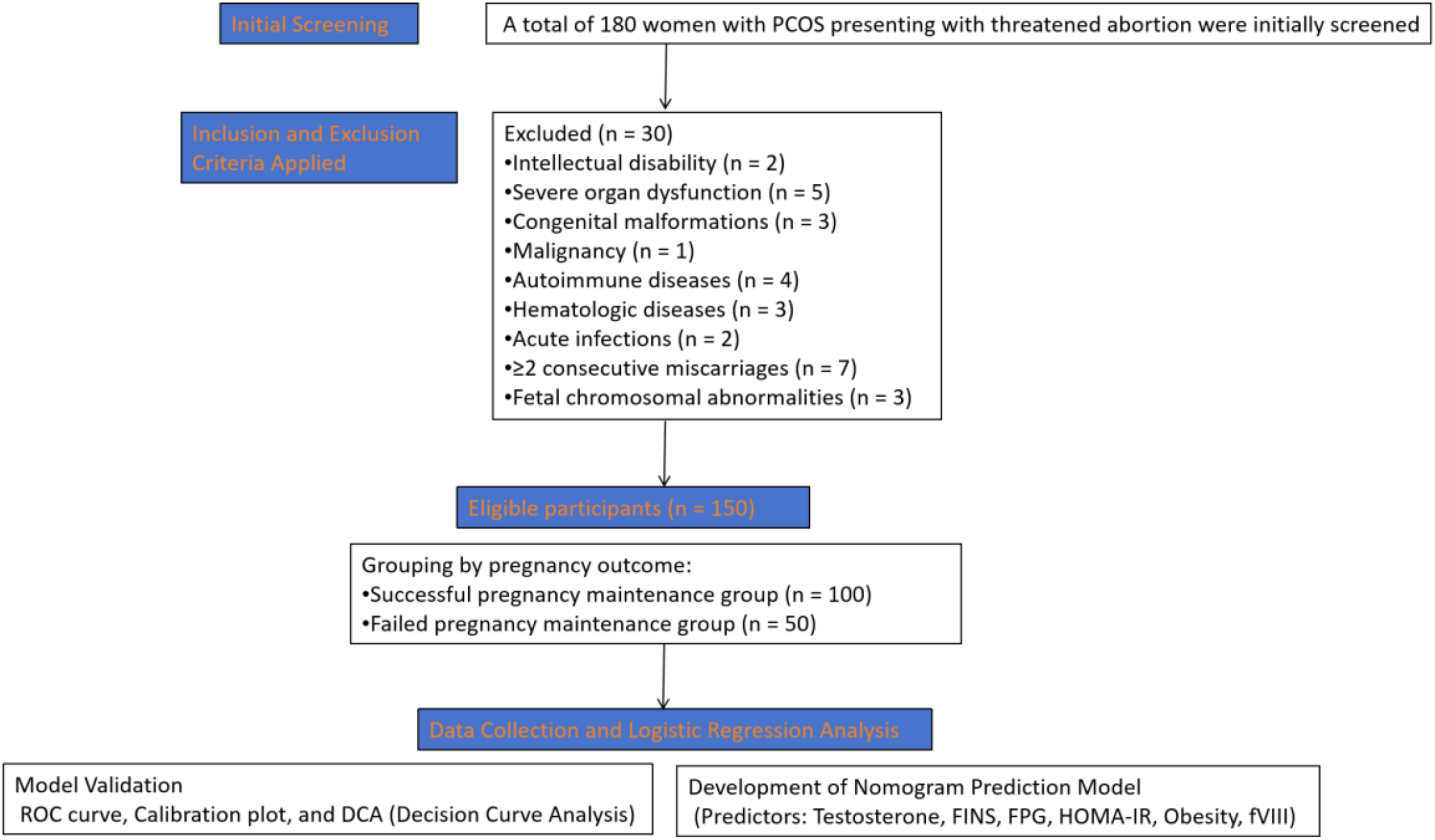
Flowchart of participant selection and analysis process. PCOS, polycystic ovary syndrome; FINS, fasting insulin; FPG, fasting plasma glucose; IR, insulin resistance; fVIII, coagulation factor VIII; SAS, Self-rating Anxiety Scale; ROC, receiver operating characteristic; AUC, area under the curve; DCA, decision curve analysis.

**Figure 1** illustrates the participant selection process. Initially, 180 PCOS patients with threatened abortion were screened for eligibility. After applying strict inclusion and exclusion criteria, 30 patients were excluded for various medical reasons including severe organ dysfunction, autoimmune diseases, recurrent miscarriage history, and fetal chromosomal abnormalities. The final analysis included 150 patients who were subsequently grouped by pregnancy outcome for comparative analysis and model development.

### Diagnostic criteria

PCOS diagnosis was established according to Revised 2003 Rotterdam Criteria (26), requiring at least two of the following: (1) polycystic ovarian morphology on ultrasound (ovarian volume >10mL or ≥12 follicles measuring 2-9mm in diameter); (2) clinical or biochemical evidence of hyperandrogenism; or (3) oligo-ovulation or anovulation. Successful pregnancy maintenance was defined as continuation of pregnancy beyond 12 weeks with confirmed fetal viability and no requirement for surgical intervention.

#### Inclusion criteria

Eligible participants met all of the following criteria: (1) diagnosis of early threatened abortion (gestational age ≤12 weeks) according to Teede’s criteria (27), presenting with vaginal bleeding/spotting (with or without abdominal pain), closed cervical os, intact gestational sac, and uterine size appropriate for gestational age; (2) confirmed singleton intrauterine pregnancy ≤12 weeks gestation; (3) availability of complete clinical and follow-up data; and (4) spontaneous conception.

#### Exclusion criteria

Patients were excluded if they presented with any of the following conditions: (1) intellectual disability or cognitive impairment; (2) severe hepatic/renal insufficiency or cardiac dysfunction (NYHA class III-IV); (3) congenital malformations of reproductive system; (4) active malignant tumors; (5) autoimmune diseases (e.g., SLE, rheumatoid arthritis) or connective tissue disorders; (6) hematologic diseases (including coagulation disorders and hemoglobinopathies); (7) acute severe infections or life-threatening conditions; (8) history of ≥2 consecutive spontaneous abortions; (9) threatened abortion caused by suspected or confirmed fetal chromosomal abnormalities. It is important to note that routine karyotype analysis of products of conception was not performed for all cases of miscarriage. This exclusion was applied based on one or more of the following criteria: (a) availability of a clinical karyotype report confirming aneuploidy from products of conception; (b) prenatal cell-free DNA testing indicating a high risk for common aneuploidies prior to miscarriage; or (c) a medical history strongly suggestive of a genetic etiology (e.g., parental balanced translocation). The majority of miscarriages were attributed to the underlying pathophysiological mechanisms of PCOS after excluding these identifiable causes; or (10) conception achieved through *in vitro* fertilization (IVF) or other assisted reproductive technologies (ART).

### Outcome

The primary outcome was failed pregnancy maintenance, defined as a spontaneous miscarriage occurring within 12 weeks of gestation following a diagnosis of threatened abortion. Successful pregnancy maintenance was defined as continuation of pregnancy beyond 12 weeks with confirmed fetal viability and no requirement for surgical intervention.

### Predictors

All candidate predictors were measured at the time of the threatened abortion diagnosis, prior to the known outcome. The predictors considered included: maternal age, PCOS duration, folic acid use, testosterone (T), fasting insulin (FINS), fasting plasma glucose (FPG), insulin resistance (IR) defined by HOMA-IR, obesity (BMI ≥25 kg/m²), total cholesterol (TC), triglycerides (TG), D-dimer (D-D), coagulation factor VIII (fVIII), and Self-Rating Anxiety Scale (SAS) score.

These variables were evaluated as candidate predictors in the initial screening; however, only testosterone, fasting insulin, and fasting plasma glucose were retained in the final multivariable model used to construct the nomogram.

### Sample size

The sample size for developing the multivariable prediction model was determined based on the widely accepted criterion of events per variable (EPV). To ensure robust and stable model estimates and minimize overfitting, a minimum EPV of 10 is recommended (28, 29).

In this study, the outcome event of interest was ‘failed pregnancy maintenance,’ which occurred in 50 out of 150 participants. In this study, the outcome event of interest was “failed pregnancy maintenance,” which occurred in 50 out of 150 participants. After re-estimating the multivariable model to avoid multicollinearity between fasting insulin, fasting plasma glucose, and HOMA-IR, our final logistic regression model incorporated 3 independent predictors: serum testosterone, fasting insulin, and fasting plasma glucose.

Thus, the effective EPV for this study was calculated as:


EPV=Number of Events/Number of Predictors=50/3≈16.7

This value exceeds the commonly recommended threshold of 10 events per variable, supporting the stability and robustness of the model estimates (28–30). The model’s performance was further rigorously evaluated and internally validated using bootstrapping to correct for any potential optimism, thereby enhancing the reliability of our findings.

### Missing data

The extent of missing data for all variables was less than 5%. We performed a complete-case analysis for model development.

### Data collection

Trained research nurses collected data using standardized electronic case report forms. Baseline demographic and clinical characteristics included maternal age (calculated from birthdate to enrollment date), gestational age at presentation, body mass index (BMI, with obesity defined as ≥25 kg/m² according to WHO criteria), parity, duration of polycystic ovary syndrome (from first diagnostic confirmation), and documented folic acid supplementation (≥400 μg/day for ≥3 months before conception).

Laboratory assessments were performed in the hospital’s CAP-accredited core laboratory. Serum testosterone was measured by electrochemiluminescence immunoassay (ECLIA; Roche Cobas e411, CV < 5%), fasting insulin by ECLIA (Roche, CV < 7%), and fasting glucose by the hexokinase method (Hitachi 7600, CV < 3%). Lipid profiles were analyzed using enzymatic assays (Hitachi 7600, CV < 5%). Coagulation studies included D-dimer (immunoturbidimetry, CV < 8%) and factor VIII activity (one-stage clotting assay, CV < 10%) on a Sysmex CS-5100 analyzer. The homeostasis model assessment of insulin resistance (HOMA-IR) was calculated as fasting insulin [μU/mL] × fasting glucose [mmol/L]/22.5, with insulin resistance defined as HOMA-IR ≥ 2.77 according to Matthews et al. (31).

Psychological status was evaluated at enrollment using the validated Chinese version of the Self-Rating Anxiety Scale (SAS; Cronbach’s α = 0.85), administered by licensed clinical psychologists.

### Statistical analysis

All analyses followed a pre-specified statistical analysis plan. Continuous variables were reported as mean ± SD (normally distributed) or median[IQR] (non-normal), verified by Shapiro-Wilk tests with Q-Q plots. Between-group comparisons used independent t-tests (normal data) or Mann-Whitney U tests (non-normal). Categorical variables were compared using χ² tests with Yates’ correction or Fisher’s exact tests for small cell counts. Model development was performed using binary logistic regression with failed pregnancy maintenance as the dependent variable. Variables showing an association with the outcome in univariate analysis (P < 0.10) were included as candidate predictors. The final model was constructed using backward stepwise selection with a retention threshold of P<0.05. In light of the mathematical dependence of HOMA-IR on FINS and FPG, we re-estimated the multivariable model to avoid multicollinearity. Specifically, HOMA-IR was not entered into the multivariable model when FINS and FPG were included; instead, only the directly measured variables (testosterone, fasting insulin, and fasting plasma glucose) were retained in the final model. Multicollinearity was assessed using variance inflation factors (VIF < 5 indicated no severe collinearity).

Model performance was comprehensively evaluated through multiple approaches: (1) discrimination (AUC with DeLong 95% CI); (2) calibration (Hosmer-Lemeshow test with 10 risk groups); and (3) clinical utility (decision curve analysis from 0-100% risk thresholds). Sensitivity analyses included multiple imputation (20 iterations) for missing data <5% and complete-case analysis. All tests were two-tailed with α=0.05, adjusted for multiple comparisons using Holm-Bonferroni method when appropriate, and analyses were conducted using R software (version 4.2.1).

The model underwent internal validation using bootstrap resampling with 1000 repetitions to correct for overoptimism. Given that the bootstrap-validated model demonstrated satisfactory calibration and discrimination, no further model updating or recalibration was deemed necessary. Furthermore, as the development and validation samples were derived from the same dataset, the distributions of all predictors and the outcome were consistent between them, indicating good internal stability of the final model.

## Results

### Comparison of clinical data

A comparative analysis of clinical factors between the two patient groups was performed. The baseline characteristics and clinical parameters of the study participants, stratified by pregnancy outcome, are summarized in **Table 1**. It revealed significant differences in the following variables: T, FINS, FPG, IR, obesity, fVIII, and SAS scores (all *P* < 0.05). In contrast, the two groups were comparable in terms of primiparity, PCOS disease duration, folic acid supplementation, TC, TG, and D-dimer levels (all *P* > 0.05). Detailed individual-level data used for model development are provided in **Supplementary Table S1**, which lists all collected variables for each participant (de-identified).

**Table 1:** Comparison of baseline characteristics and clinical parameters between the successful and failed pregnancy maintenance groups.

Index	Successful group(n=100)	Unsuccessful group(n=50)	P
Maternal Age (years)	30.62 ± 6.57	30.53 ± 6.71	0.939
Gestational Age (weeks)	7.54 ± 1.85	7.42 ± 1.94	0.714
Primipara	75	43	0.121
PCOS disease course(years)	4.25 ± 1.56	4.12 ± 1.51	0.628
Folic acid supplement	68	32	0.624
T(ng/mL)	31.03 ± 11.54	58.75 ± 12.33	0.000
FINS (μIU/mL)	8.59 ± 2.16	12.01 ± 2.81	0.000
FPG (mmol/L)	5.38 ± 0.36	5.87 ± 0.75	0.000
IR (n)	14	28	0.000
Obesity (kg/m)			0.002
<25	67	20	
≥25	33	30	
TC (mmol/L)	5.21 ± 0.41	5.27 ± 0.43	0.407
TG (mmol/L)	1.37 ± 0.28	1.41 ± 0.31	0.428
D-D (mg/L)	0.14 ± 0.04	0.15 ± 0.05	0.187
fVIII (%)	72.25 ± 9.43	76.81 ± 8.62	0.005
SAS			0.000
<40	63	16	
≥40	37	34	

*T, Testosterone; FINS, fasting insulin; FPG, fasting blood glucose; HOMA-IR, Insulin resistance index; TC, total cholesterol; TG, triglyceride; D-D, D-Dimer; fVIII, coagulation factor VIII; SAS, Self-rating Anxiety Scale.

### Construction and evaluation of the prognostic nomogram model

**Figure 2:**
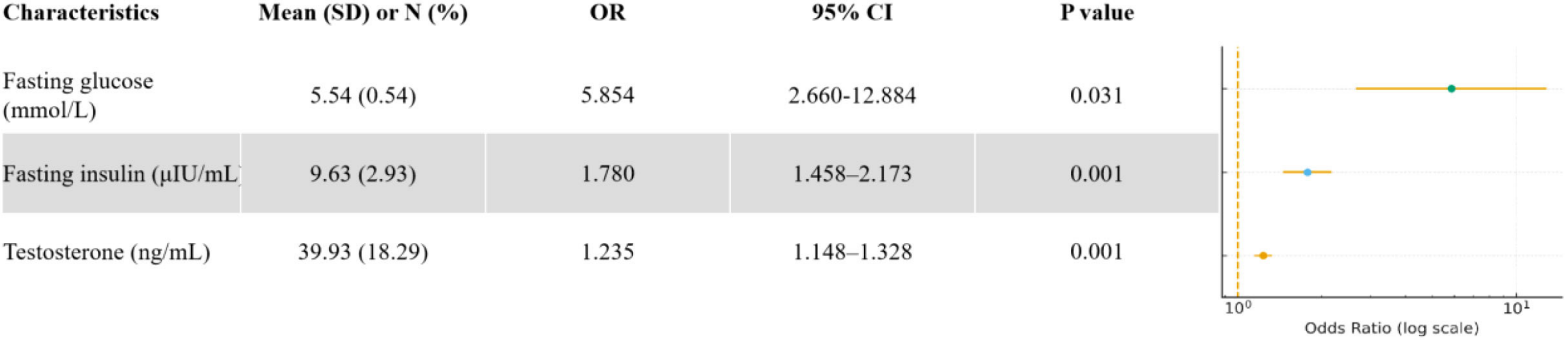
Forest plot of multivariable logistic regression analysis showing predictors of failed pregnancy maintenance in women with polycystic ovary syndrome (PCOS). Odds ratios (ORs) with 95% confidence intervals (CIs) are shown for each predictor, together with the corresponding P values.

**Figure 3:**
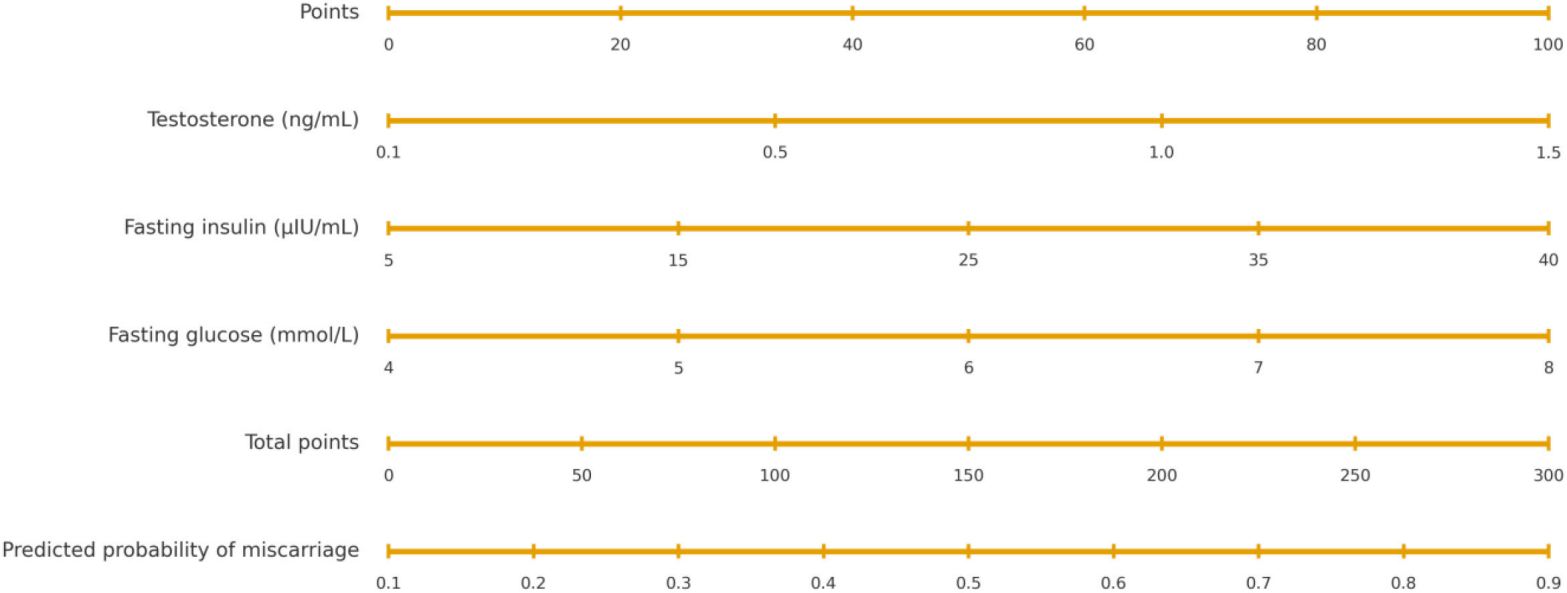
Nomogram for predicting the risk of failed pregnancy maintenance in women with polycystic ovary syndrome (PCOS) presenting with threatened abortion. The nomogram incorporates three predictors: serum testosterone, fasting insulin, and fasting plasma glucose. For each variable, a vertical line is drawn to the “Points” scale to obtain a score; the scores are then summed to obtain the “Total points”, which correspond to the predicted risk of miscarriage.

Variables with P < 0.10 in univariate analysis were entered into a multivariable logistic regression model. Because HOMA-IR is mathematically derived from fasting insulin and fasting plasma glucose, it was not entered together with its components to avoid multicollinearity. After stepwise selection, three independent predictors remained significantly associated with failed pregnancy maintenance in women with PCOS presenting with threatened abortion: serum testosterone, fasting insulin, and fasting glucose (**Figure 2**). These three predictors were incorporated into a predictive nomogram (**Figure 3**) to estimate the individualized probability of miscarriage.

Although coagulation factor VIII activity and SAS score showed significant differences between the two outcome groups in univariate analyses (**Table 1**), they did not remain statistically significant in the multivariable logistic regression model (factor VIII: P = 0.059; SAS: P = 0.277) and were therefore not included as independent predictors in the final nomogram.

**Figure 4:**
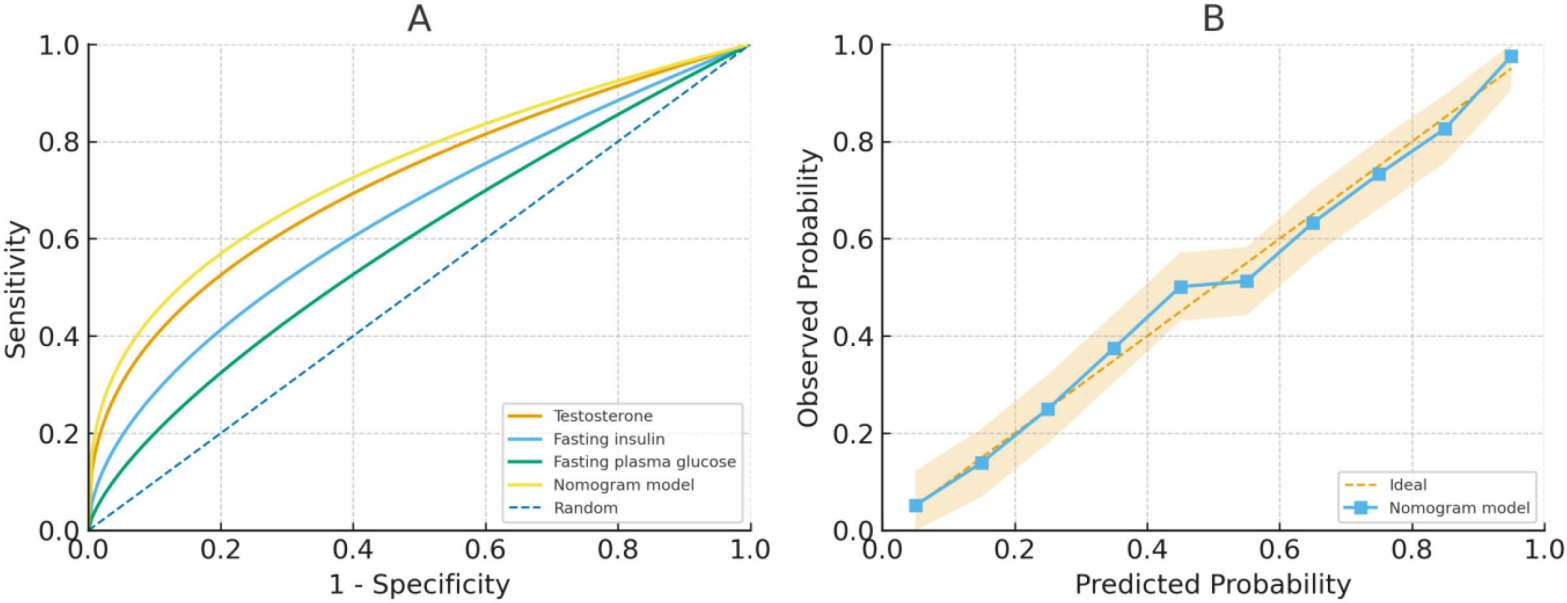
Receiver-operating-characteristic (ROC) and calibration curves for miscarriage prediction in women with PCOS presenting with threatened abortion. **(A)** ROC curves comparing the discriminative performance of individual predictors (testosterone, fasting insulin, and fasting plasma glucose) and the overall nomogram model for predicting failed pregnancy maintenance. **(B)** Calibration curve of the nomogram model showing good agreement between predicted and observed probabilities of miscarriage, indicating satisfactory calibration and clinical applicability.

The model’s discriminative performance was evaluated by ROC analysis (**Figure 4A**). Among the individual predictors included in the final model, testosterone exhibited the highest diagnostic value for predicting failed pregnancy maintenance, whereas the combined nomogram achieved the best overall discrimination. The calibration curve (**Figure 4B**) further showed good agreement between predicted and observed probabilities, supporting the clinical utility of the nomogram for individualized risk assessment in early threatened abortion.

### Calibration curve and DCA curve

**Figure 5:**
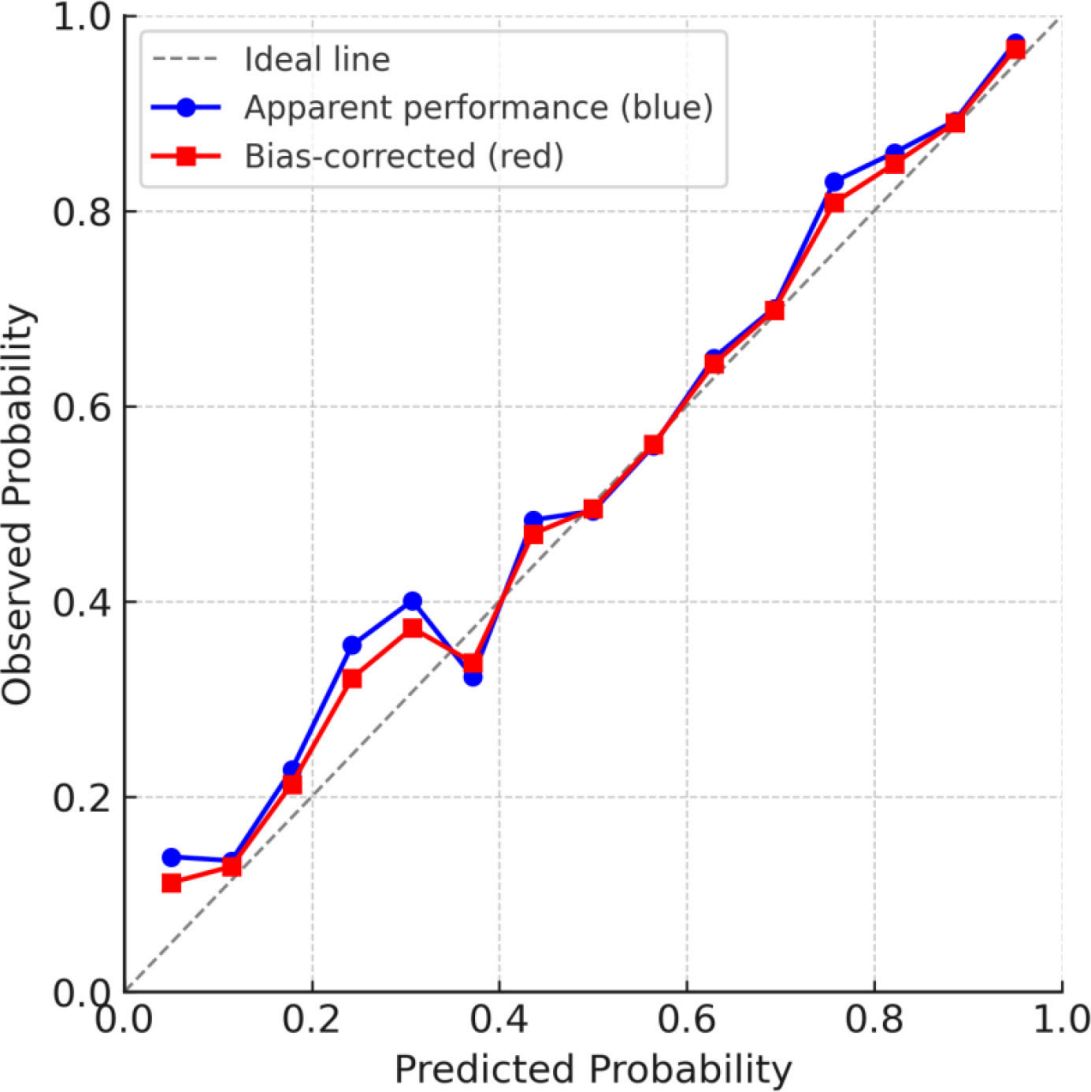
Calibration curve of the nomogram predicting miscarriage risk in women with PCOS presenting with threatened abortion. The blue line represents the apparent performance of the model, the red line represents the bias-corrected performance after bootstrapping, and the dashed gray line represents the ideal reference line.

**Figure 6:**
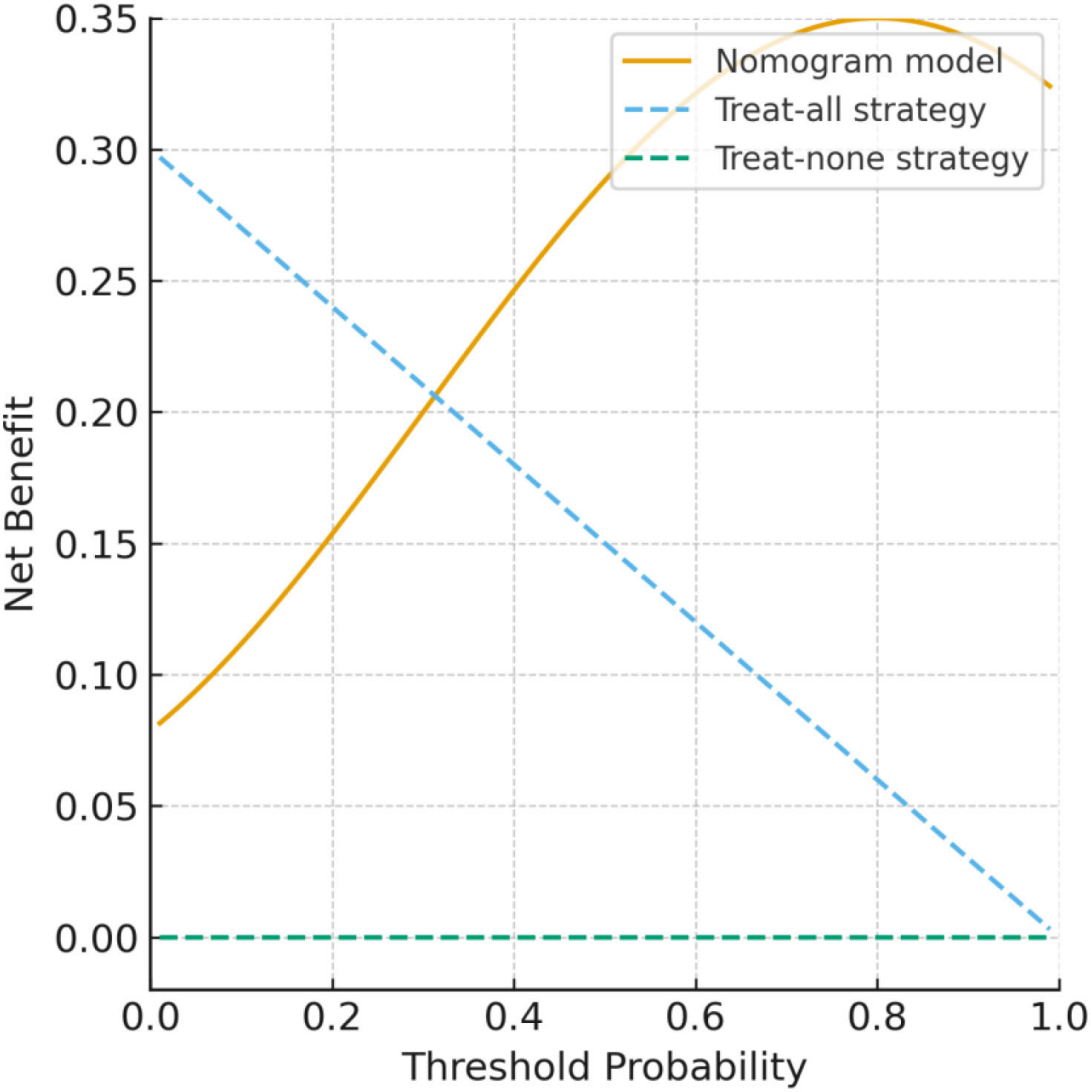
Decision curve analysis (DCA) of the nomogram model for predicting miscarriage risk in PCOS patients with threatened abortion. The model demonstrates a higher net clinical benefit across a wide range of threshold probabilities compared with the “treat-all” and “treat-none” strategies.

The predictive performance of the model was assessed using ROC analysis, which yielded an AUC ranging from 0.635 to 0.955, indicating strong discriminative ability. Calibration curve for the nomogram model demonstrated excellent agreement between predicted and observed outcomes, closely approximating the ideal curve (**Figure 5**). Furthermore, decision curve analysis (DCA) was conducted to evaluate clinical utility (**Figure 6**). The model’s net benefit curve consistently surpassed those of the extreme scenarios (treat-all and treat-none strategies), underscoring its robust clinical applicability.

## Discussion

PCOS is a prevalent endocrine disorder characterized by heterogeneous manifestations, including hyperandrogenism, IR, and ovulatory dysfunction (1, 2). A significant clinical challenge in managing PCOS is the markedly increased risk of miscarriage, which persists even after conception occurs (12, 13). TA in these patients often precedes pregnancy loss, with rates significantly exceeding those in the non-PCOS population. While progesterone supplementation remains the cornerstone of TA management, a substantial proportion of PCOS patients experience therapeutic failure, underscoring the limitations of a one-size-fits-all approach and the need for personalized risk stratification (19, 20). Our study addressed this critical gap by developing and validating a nomogram model that integrates key pathophysiological markers to predict the risk of miscarriage in PCOS patients presenting with TA. The final model unequivocally identifies serum testosterone, fasting insulin, and fasting glucose as independent predictors, offering a refined tool for clinical decision-making and shedding light on the predominant mechanisms driving pregnancy loss in this high-risk cohort.

In our model, serum testosterone emerged as the most potent predictor of failed pregnancy maintenance. This finding powerfully extends the established link between hyperandrogenism and general miscarriage risk in PCOS (32, 33). It specifically indicates that among patients already diagnosed with TA and receiving standard progesterone support, elevated testosterone levels are a critical marker of likely therapeutic failure. The underlying mechanism likely involves androgen-mediated impairment of endometrial receptivity. Elevated testosterone can disrupt the process of decidualization and downregulate progesterone receptor signaling, effectively creating a state of “progesterone resistance” (8, 10). In such an environment, the endometrium fails to mount an adequate response to exogenous progesterone supplementation, leading to inadequate placental embedding and eventual pregnancy loss (34). This pathophysiological insight explains why luteal phase support alone may be insufficient for a subset of PCOS patients with significant hyperandrogenism, highlighting the need for strategies that directly address or circumvent this androgen excess.

Concurrently, our results confirm that dysregulated maternal metabolism, represented by elevated fasting insulin and fasting glucose, is a cornerstone of adverse outcomes following TA. While progesterone addresses luteal phase deficiency, it does not counteract the detrimental effects of hyperinsulinemia and hyperglycemia at the maternal-fetal interface. Insulin resistance and compensatory hyperinsulinemia can promote a pro-inflammatory and pro-oxidant state, directly damaging trophoblast cells and impairing their invasive capacity, which is crucial for placentation (9, 16). This metabolic milieu fundamentally compromises the foundational process of placental development, leading to progressive placental insufficiency and miscarriage, despite initial progesterone support (15). The independent predictive value of these metabolic markers underscores a critical limitation of conventional TA management protocols that do not actively address underlying IR and glycemic instability, pointing to the potential utility of adjunctive insulin-sensitizing therapies.

It is noteworthy that other factors, such as obesity, coagulation factor VIII, and maternal anxiety, which demonstrated significant differences in our univariate analysis, were not retained as independent predictors in the final multivariable model. This suggests that their apparent association with miscarriage risk may be mediated through or confounded by the core endocrine and metabolic disturbances captured by testosterone, insulin, and glucose. For instance, obesity is a well-known amplifier of both hyperandrogenism and IR (35), and its effect may be largely accounted for by these more proximate physiological derangements. Similarly, the prothrombotic state associated with elevated factor VIII is often exacerbated by the IR present in PCOS (17, 18), and its contribution may not be independent of the metabolic dysregulation. While these factors remain clinically relevant components of the PCOS phenotype, our model indicates that the direct pathophysiological drivers of miscarriage in the context of TA are hyperandrogenism and dysglycemia.

A pivotal strength of this study is the development and validation of the first integrated nomogram that synthesizes key endocrine and metabolic markers to predict miscarriage risk in PCOS patients presenting with TA. The model’s robust performance, evidenced by strong discrimination, satisfactory calibration, and superior clinical utility on decision curve analysis, underscores its potential for immediate clinical application. Furthermore, conducting this research within a cohort where all patients received a consistent, standard-of-care treatment protocol primarily based on progesterone supplementation crucially enhances internal validity. This design demonstrates that the identified risk factors predict outcome despite uniform treatment, highlighting their power as independent prognostic markers.

The clinical utility of our model is magnified by the finding that a significant proportion of pregnancies miscarried despite standard care. This starkly highlights the existence of distinct pathophysiological pathways that lead to therapeutic failure and the necessity for a more nuanced management strategy. Our nomogram provides a framework for such personalized intervention. For instance: 1) Patients flagged primarily for hyperandrogenism (high testosterone) might be candidates for more potent or alternative forms of luteal support to overcome potential endometrial progesterone resistance. 2) Those with prominent metabolic dysregulation (high fasting insulin and glucose) could be targeted for adjunctive metformin therapy to address the underlying insulin resistance that progesterone does not mitigate (19, 20). By enabling early identification of high-risk patients, the model facilitates timely and targeted interventions, moving beyond a reactive approach to a proactive, precision-based strategy.

However, the interpretation of our findings must be tempered by an acknowledgment of the study’s limitations. The single-center, retrospective design inherently carries risks of selection bias and limits the immediate generalizability of our findings. The sample size, while adequate for initial model development, is modest. Future multi-center, prospective studies with larger sample sizes are imperative for external validation and refinement of the model. Additionally, despite incorporating a wide array of clinical variables, the influence of unmeasured confounders cannot be entirely ruled out.

Notwithstanding these limitations, this study presents a novel and promising tool that translates the complex pathophysiology of PCOS into a clinically actionable prognostic instrument. By pinpointing hyperandrogenism and metabolic dysregulation as the key drivers of miscarriage risk in PCOS patients with TA, our nomogram paves the way for a more effective and personalized management strategy, ultimately aiming to improve pregnancy outcomes in this vulnerable population.

## Conclusion

In conclusion, this study successfully develops and validates a novel nomogram model for predicting the risk of miscarriage in PCOS patients presenting with threatened abortion. The model definitively identifies elevated serum testosterone, fasting insulin, and fasting glucose as key independent predictors of failed pregnancy maintenance. These findings underscore that hyperandrogenism and profound metabolic dysfunction are the predominant drivers of miscarriage in this high-risk population, even in the context of standard progesterone support.

The clinical significance of this model lies in its ability to move beyond a uniform treatment approach. By providing a quantifiable, individualized risk assessment at the time of threatened abortion diagnosis, it enables clinicians to stratify patients and identify those who may benefit from intensified, personalized interventions. Such interventions could include more aggressive luteal phase support for hyperandrogenism or the adjunctive use of insulin-sensitizing agents like metformin for significant metabolic dysregulation.

Therefore, the integration of this nomogram into clinical practice holds the potential to facilitate earlier, more targeted therapeutic strategies, ultimately aiming to mitigate the risk of miscarriage and improve pregnancy outcomes for women with PCOS. Future multicenter, prospective studies are warranted to further validate and refine this tool for broader application.

## Data Availability

The original contributions presented in the study are included in the article/**Supplementary Material**. Further inquiries can be directed to the corresponding author.
