# Supplementary material for: Predictors of miscarriage in polycystic ovary syndrome patients with threatened abortion: development and validation of a nomogram model
Source: Front Endocrinol (Lausanne). 2026 Jan 9;16:1689878. doi: 10.3389/fendo.2025.1689878 (PMC12827140; doi:10.3389/fendo.2025.1689878)
Supplement: Supplementary file 1 [file Table1.docx]

**Supplementary Table S1.** Raw de-identified dataset including baseline demographic, clinical, laboratory, and psychological parameters for all 150 participants.

| **ID** | **Outcome** | **Maternal_Age_yrs** | **Gestational_Age_wks** | **BMI_kg_m2** | **Primipara** | **PCOS_Duration_yrs** | **Folic_Acid_Use** | **Testosterone_ng_mL** | **Fasting_Insulin_uIU_mL** | **Fasting_Glucose_mmol_L** | **HOMA_IR** | **Total_Cholesterol_mmol_L** | **Triglycerides_mmol_L** | **D_dimer_mg_L** | **Factor_VIII_percent** | **SAS_score** | **Insulin_Resistance_flag** | **Obesity_flag** | **SAS_ge_40_flag** |
| --- | --- | --- | --- | --- | --- | --- | --- | --- | --- | --- | --- | --- | --- | --- | --- | --- | --- | --- | --- |
| 001 | 0 | 33.88 | 4.92 | 25.24 | 1 | 2.96 | Y | 12.63 | 10.59 | 5.653 | 2.66 | 4.04 | 0.88 | 0.155 | 67.32 | 39.6 | N | N | Y |
| 002 | 0 | 29.71 | 6.76 | 25.89 | 1 | 3.38 | N | 24.11 | 12.71 | 5.048 | 2.85 | 4.06 | 1.01 | 0.124 | 82.14 | 30.9 | Y | N | N |
| 003 | 0 | 34.88 | 6.91 | 27.57 | 1 | 5.42 | Y | 31.09 | 5.57 | 5.693 | 1.41 | 4.17 | 1.26 | 0.141 | 65.61 | 34.6 | N | Y | N |
| 004 | 0 | 40.63 | 6.06 | 27.47 | 1 | 5.2 | N | 31.57 | 9.81 | 5.868 | 2.56 | 4.43 | 1.4 | 0.191 | 58.97 | 31.2 | N | Y | N |
| 005 | 0 | 29.08 | 7.24 | 19.69 | 1 | 4.22 | N | 25.84 | 7.18 | 5.529 | 1.77 | 3.88 | 1.2 | 0.148 | 57.57 | 31.4 | Y | N | N |
| 006 | 0 | 29.08 | 8.29 | 21.1 | 1 | 4.43 | Y | 38.22 | 7.54 | 6.056 | 2.03 | 4.49 | 0.93 | 0.142 | 77.96 | 32.1 | N | N | N |
| 007 | 0 | 41 | 11.03 | 25.75 | 0 | 6.24 | Y | 18.71 | 7.31 | 5.101 | 1.66 | 4.12 | 0.66 | 0.086 | 60.18 | 35.3 | N | N | N |
| 008 | 0 | 35.66 | 7.86 | 25.74 | 1 | 3.33 | N | 29.39 | 6.72 | 4.932 | 1.47 | 4.56 | 1.05 | 0.17 | 88.8 | 31.1 | N | N | N |
| 009 | 0 | 27.54 | 8.02 | 25.75 | 1 | 5.1 | N | 32.42 | 8.69 | 4.74 | 1.83 | 4.52 | 1.15 | 0.166 | 52.62 | 41.5 | N | N | Y |
| 010 | 0 | 34.18 | 7.4 | 36.43 | 1 | 3.93 | Y | 36.97 | 6.8 | 5.919 | 1.79 | 4.13 | 0.7 | 0.227 | 88.25 | 28.6 | N | Y | N |
| 011 | 0 | 27.58 | 4 | 25.93 | 0 | 3.91 | Y | 39.24 | 9.17 | 5.616 | 2.29 | 3.63 | 0.45 | 0.128 | 74.24 | 32.9 | N | Y | N |
| 012 | 0 | 27.56 | 7.49 | 27.73 | 1 | 5.96 | Y | 18.05 | 8.48 | 5.36 | 2.02 | 3.97 | 1.67 | 0.149 | 71.34 | 31.4 | Y | Y | N |
| 013 | 0 | 32.21 | 7.65 | 27.15 | 1 | 5.54 | Y | 13.33 | 8.07 | 5.481 | 1.97 | 3.68 | 0.44 | 0.15 | 67.11 | 42.7 | N | Y | Y |
| 014 | 0 | 18.05 | 12 | 26.18 | 0 | 5.52 | Y | 45.77 | 6.63 | 4.975 | 1.47 | 4.59 | 1.69 | 0.203 | 76.01 | 35.2 | N | Y | N |
| 015 | 0 | 19.29 | 7.18 | 23.09 | 0 | 6.29 | N | 34.86 | 7.34 | 6.26 | 2.04 | 4.32 | 1.17 | 0.136 | 71.9 | 40.2 | N | N | Y |
| 016 | 0 | 26.93 | 8.1 | 26.53 | 1 | 4.28 | Y | 22.39 | 10.22 | 5.427 | 2.47 | 4.08 | 0.65 | 0.151 | 82.65 | 25.1 | N | Y | N |
| 017 | 0 | 23.97 | 7.48 | 21.63 | 1 | 5.31 | Y | 48.93 | 9.67 | 5.419 | 2.33 | 3.62 | 1.02 | 0.164 | 73.33 | 35.6 | Y | N | N |
| 018 | 0 | 32.68 | 5.38 | 23.34 | 1 | 3.77 | Y | 32.36 | 6.48 | 5.641 | 1.62 | 4 | 1.07 | 0.147 | 73.67 | 39.3 | Y | N | Y |
| 019 | 0 | 24.65 | 9.65 | 22.55 | 1 | 4.76 | Y | 44.64 | 8.8 | 5.553 | 2.17 | 4.53 | 0.95 | 0.122 | 68.82 | 34.5 | N | N | N |
| 020 | 0 | 21.34 | 8.93 | 24.36 | 1 | 4.05 | Y | 31.81 | 10.21 | 5.461 | 2.48 | 4.54 | 0.96 | 0.148 | 71.71 | 40.4 | N | N | Y |
| 021 | 0 | 40.25 | 9 | 31.51 | 1 | 4.4 | N | 54.81 | 4.98 | 5.095 | 1.13 | 4.62 | 1.12 | 0.183 | 75.15 | 30.9 | N | Y | N |
| 022 | 0 | 29.14 | 5.86 | 18.12 | 0 | 5.18 | Y | 51.29 | 9.76 | 5.55 | 2.41 | 4.03 | 1.12 | 0.099 | 56.12 | 42.5 | N | N | Y |
| 023 | 0 | 31.06 | 10.14 | 26.3 | 0 | 2.97 | N | 28.16 | 7.16 | 6.058 | 1.93 | 4.08 | 1.13 | 0.145 | 59.54 | 47.8 | N | Y | Y |
| 024 | 0 | 21.26 | 4.95 | 18.94 | 0 | 7.51 | Y | 42.24 | 9.82 | 5.864 | 2.56 | 4.5 | 1.08 | 0.112 | 79.26 | 31.8 | N | N | N |
| 025 | 0 | 27.04 | 8.63 | 22.59 | 1 | 2.68 | Y | 38.48 | 6.94 | 5.954 | 1.84 | 4.05 | 0.82 | 0.188 | 73.86 | 31.3 | N | N | N |
| 026 | 0 | 31.35 | 11.59 | 27.58 | 1 | 2.36 | Y | 46.82 | 4.69 | 5.196 | 1.08 | 4.43 | 1.19 | 0.079 | 70.52 | 42.7 | N | Y | Y |
| 027 | 0 | 23.06 | 5.71 | 24.31 | 1 | 6.06 | Y | 19.89 | 5.07 | 5.024 | 1.13 | 3.73 | 1.31 | 0.118 | 72.42 | 43.5 | N | N | Y |
| 028 | 0 | 33.09 | 6.49 | 20.65 | 0 | 5.48 | Y | 38.95 | 8.69 | 5.335 | 2.06 | 3.72 | 1.36 | 0.155 | 75.53 | 30.9 | N | N | N |
| 029 | 0 | 26.67 | 7.72 | 21.81 | 1 | 5.22 | N | 43.24 | 9.15 | 5.4 | 2.2 | 4.48 | 1.2 | 0.203 | 67.16 | 31.5 | N | N | N |
| 030 | 0 | 28.7 | 6.61 | 26.27 | 1 | 5.23 | N | 10.73 | 6.64 | 5.774 | 1.7 | 4.05 | 1.24 | 0.137 | 64.91 | 32.3 | N | Y | N |
| 031 | 0 | 26.67 | 4.67 | 21.76 | 1 | 4.23 | N | 17.38 | 9.97 | 4.771 | 2.11 | 4.63 | 1.25 | 0.118 | 74.1 | 25.9 | N | N | N |
| 032 | 0 | 42.79 | 7.67 | 24.79 | 1 | 2.85 | N | 7.5 | 5 | 5.931 | 1.32 | 3.93 | 1.17 | 0.215 | 63.02 | 28.5 | N | N | N |
| 033 | 0 | 30.53 | 5.57 | 24.25 | 0 | 4.37 | Y | 27.92 | 8.45 | 5.323 | 2 | 4.65 | 1.65 | 0.082 | 76.1 | 28 | N | N | N |
| 034 | 0 | 23.67 | 8.42 | 22.01 | 1 | 3.19 | N | 39.31 | 5.97 | 5.226 | 1.39 | 4.12 | 1.31 | 0.052 | 56.19 | 29.4 | N | N | N |
| 035 | 0 | 36.02 | 5.84 | 30.96 | 1 | 5.77 | N | 48.37 | 7.18 | 5.016 | 1.6 | 4.65 | 0.9 | 0.158 | 81.95 | 33.8 | Y | Y | N |
| 036 | 0 | 22.6 | 10.41 | 26.13 | 1 | 4.02 | N | 31.89 | 8.69 | 4.784 | 1.85 | 4.91 | 0.83 | 0.12 | 76.71 | 35.4 | N | Y | N |
| 037 | 0 | 31.99 | 6.09 | 17.62 | 1 | 2.96 | Y | 49.82 | 6.73 | 5.676 | 1.7 | 4.52 | 1.18 | 0.099 | 74.66 | 43.3 | N | N | Y |
| 038 | 0 | 18 | 6.94 | 24.7 | 0 | 3.75 | Y | 15.1 | 7.76 | 5.406 | 1.86 | 4.22 | 1.74 | 0.168 | 81.52 | 28 | N | N | N |
| 039 | 0 | 21.89 | 9.05 | 21.98 | 1 | 4.89 | N | 11.37 | 10.76 | 4.916 | 2.35 | 4.08 | 1.02 | 0.15 | 87.96 | 39.9 | N | N | Y |
| 040 | 0 | 31.91 | 5.26 | 26.83 | 0 | 3.37 | Y | 30.39 | 7.34 | 4.914 | 1.6 | 4.73 | 1.29 | 0.117 | 81.82 | 32.7 | N | Y | N |
| 041 | 0 | 35.47 | 7.96 | 21.56 | 0 | 2.97 | Y | 35.46 | 10.4 | 5.259 | 2.43 | 4.71 | 1.13 | 0.089 | 54.89 | 33.7 | Y | N | N |
| 042 | 0 | 31.75 | 9.96 | 23.73 | 1 | 4.63 | N | 30.65 | 6.15 | 5.981 | 1.63 | 4.33 | 1.58 | 0.175 | 60.18 | 38 | N | N | Y |
| 043 | 0 | 29.86 | 4.57 | 25.72 | 1 | 4.63 | Y | 7.17 | 9.73 | 5.287 | 2.29 | 3.87 | 1.07 | 0.166 | 66.36 | 27.3 | N | N | N |
| 044 | 0 | 28.64 | 7.88 | 26.87 | 1 | 3.46 | Y | 30 | 11.7 | 4.839 | 2.52 | 4.52 | 1.05 | 0.136 | 72.5 | 36.3 | N | Y | N |
| 045 | 0 | 20.91 | 8.02 | 20.26 | 1 | 3.52 | Y | 15.98 | 3.25 | 5.292 | 0.76 | 3.97 | 1.25 | 0.214 | 77.13 | 35 | Y | N | N |
| 046 | 0 | 25.89 | 8.99 | 23.03 | 1 | 4.61 | Y | 38.76 | 6.87 | 5.282 | 1.61 | 4.73 | 1.06 | 0.097 | 65.41 | 37 | N | N | N |
| 047 | 0 | 27.59 | 5.25 | 22.58 | 1 | 1.99 | N | 35.26 | 9.84 | 4.409 | 1.93 | 4.13 | 0.94 | 0.079 | 74.01 | 35.7 | Y | N | N |
| 048 | 0 | 37.57 | 5.1 | 22.01 | 1 | 2.05 | Y | 20.18 | 8.15 | 5.36 | 1.94 | 4.08 | 1.37 | 0.112 | 65.13 | 48.7 | N | N | Y |
| 049 | 0 | 32.88 | 8.51 | 29.75 | 1 | 3.13 | Y | 25.1 | 9.39 | 5.297 | 2.21 | 4.23 | 0.9 | 0.138 | 66.48 | 30.2 | N | Y | N |
| 050 | 0 | 19.04 | 8.09 | 25.4 | 1 | 3.92 | Y | 18.81 | 7.29 | 5.631 | 1.82 | 4.3 | 1.36 | 0.15 | 58.99 | 30.8 | Y | N | N |
| 051 | 0 | 32.75 | 8 | 20.07 | 0 | 4.74 | Y | 30.31 | 8.78 | 6.046 | 2.36 | 3.98 | 0.77 | 0.13 | 63.54 | 30.3 | N | N | N |
| 052 | 0 | 28.09 | 8.18 | 27.04 | 1 | 6.55 | Y | 42.05 | 8.25 | 5.786 | 2.12 | 5.08 | 0.76 | 0.154 | 59.5 | 30.7 | Y | Y | N |
| 053 | 0 | 26.17 | 6.28 | 30.89 | 1 | 5.59 | Y | 19.65 | 11.11 | 5.283 | 2.61 | 4.06 | 1.16 | 0.09 | 63.05 | 30.2 | N | Y | N |
| 054 | 0 | 34.64 | 7.97 | 27.4 | 1 | 4 | Y | 36.85 | 9.14 | 4.982 | 2.02 | 4.09 | 1.08 | 0.198 | 82.19 | 41.1 | N | Y | Y |
| 055 | 0 | 37.39 | 8.08 | 19.24 | 1 | 4.22 | Y | 24.91 | 9.32 | 6.306 | 2.61 | 4.43 | 0.5 | 0.137 | 63.3 | 42.5 | N | N | Y |
| 056 | 0 | 36.74 | 6.22 | 22.55 | 0 | 2.69 | N | 21.88 | 7.7 | 5.401 | 1.85 | 3.69 | 1.11 | 0.185 | 97.07 | 30.6 | N | N | N |
| 057 | 0 | 25.11 | 10.99 | 28.15 | 1 | 4.22 | N | 29.79 | 7.54 | 5.385 | 1.8 | 4.04 | 1.13 | 0.154 | 76.9 | 29 | N | Y | N |
| 058 | 0 | 28.59 | 8.42 | 21.84 | 0 | 3.8 | Y | 19.08 | 7.66 | 5.371 | 1.83 | 4.29 | 0.57 | 0.158 | 73.99 | 36.8 | N | N | N |
| 059 | 0 | 32.8 | 5.34 | 25.52 | 0 | 4.75 | Y | 24.64 | 9.44 | 5.451 | 2.29 | 4.71 | 0.98 | 0.163 | 64.16 | 30.7 | N | N | N |
| 060 | 0 | 37.03 | 8.75 | 26.58 | 1 | 2.96 | Y | 17.21 | 7.68 | 5.328 | 1.82 | 3.85 | 1.15 | 0.158 | 78.85 | 37.8 | N | Y | Y |
| 061 | 0 | 27.47 | 5.74 | 21.13 | 1 | 5.06 | N | 53.7 | 9.22 | 5.173 | 2.12 | 4.24 | 1.4 | 0.166 | 66.82 | 35.2 | N | N | N |
| 062 | 0 | 29.4 | 9 | 23.91 | 1 | 6.64 | N | 31.44 | 13.07 | 5.183 | 3.01 | 4.31 | 1.39 | 0.193 | 73.4 | 24.9 | N | N | N |
| 063 | 0 | 23.35 | 9.68 | 16 | 1 | 4.08 | Y | 22.96 | 10.47 | 5.368 | 2.5 | 3.82 | 1.14 | 0.148 | 96.39 | 43.3 | N | N | Y |
| 064 | 0 | 22.76 | 6.02 | 20.82 | 1 | 4.88 | Y | 33.5 | 7.89 | 5.184 | 1.82 | 3.75 | 1.12 | 0.168 | 71.34 | 44.8 | N | N | Y |
| 065 | 0 | 35.96 | 9.32 | 23.29 | 1 | 5.33 | Y | 29.73 | 11.18 | 5.123 | 2.55 | 4.15 | 1.06 | 0.136 | 83.09 | 30.3 | Y | N | N |
| 066 | 0 | 39.53 | 8.3 | 20.11 | 1 | 3.62 | Y | 28.48 | 7.71 | 5.418 | 1.86 | 4.14 | 1.28 | 0.198 | 65.62 | 31.7 | N | N | N |
| 067 | 0 | 30.15 | 9.06 | 29.32 | 1 | 4.6 | Y | 38.12 | 4.19 | 5.288 | 0.98 | 3.56 | 0.88 | 0.113 | 71.92 | 35.7 | Y | Y | N |
| 068 | 0 | 37.21 | 11.05 | 19.52 | 1 | 4.27 | Y | 39.77 | 6.41 | 5.921 | 1.69 | 4.18 | 0.81 | 0.212 | 88.95 | 36 | N | N | N |
| 069 | 0 | 33 | 7.09 | 22.69 | 1 | 4.4 | Y | 24.91 | 4.55 | 4.426 | 0.89 | 4.22 | 1.09 | 0.138 | 66.34 | 38 | N | N | Y |
| 070 | 0 | 26.38 | 6.15 | 24.52 | 0 | 3.04 | Y | 24.39 | 7.83 | 5.773 | 2.01 | 3.64 | 0.74 | 0.083 | 89.34 | 46.1 | N | N | Y |
| 071 | 0 | 32.99 | 5.89 | 28.71 | 1 | 4.29 | Y | 27.86 | 8.63 | 5.829 | 2.24 | 3.15 | 0.79 | 0.145 | 78.92 | 32.9 | N | Y | N |
| 072 | 0 | 40.72 | 6.03 | 19.51 | 1 | 5.03 | N | 4.47 | 12.21 | 4.634 | 2.51 | 4.21 | 1.14 | 0.113 | 66.95 | 29.2 | N | N | N |
| 073 | 0 | 30.38 | 7.4 | 27.82 | 1 | 6.51 | N | 13.54 | 9.3 | 5.257 | 2.17 | 4.28 | 0.91 | 0.174 | 78.21 | 25.7 | N | Y | N |
| 074 | 0 | 40.9 | 8.17 | 24.13 | 1 | 5.75 | Y | 46.8 | 8.12 | 5.246 | 1.89 | 4.19 | 1.2 | 0.114 | 81.42 | 29.6 | N | N | N |
| 075 | 0 | 18 | 8.05 | 20.96 | 1 | 7.61 | Y | 50.01 | 10.38 | 4.873 | 2.25 | 3.69 | 1.13 | 0.122 | 78.11 | 33.8 | N | N | N |
| 076 | 0 | 36.02 | 9.07 | 25.58 | 0 | 3.05 | Y | 28.16 | 3.81 | 5.1 | 0.86 | 4.46 | 0.98 | 0.064 | 57.44 | 44.8 | N | N | Y |
| 077 | 0 | 31.19 | 7.56 | 24.74 | 1 | 5.61 | Y | 37.68 | 9.1 | 4.98 | 2.01 | 4.62 | 0.98 | 0.122 | 65.39 | 30.9 | N | N | N |
| 078 | 0 | 28.66 | 10.23 | 22.18 | 1 | 4.54 | Y | 34.62 | 10.26 | 6.011 | 2.74 | 3.86 | 1.24 | 0.05 | 69.92 | 35.3 | N | N | N |
| 079 | 0 | 31.22 | 7.05 | 24.32 | 1 | 7.67 | Y | 66.56 | 5.4 | 5.717 | 1.37 | 5.01 | 1.12 | 0.077 | 71.55 | 33.9 | Y | N | N |
| 080 | 0 | 18 | 12 | 22.87 | 0 | 2.99 | Y | 43.95 | 11.06 | 5.838 | 2.87 | 4.45 | 0.97 | 0.17 | 78.1 | 41.1 | N | N | Y |
| 081 | 0 | 29.18 | 8.7 | 24.46 | 1 | 2.94 | N | 29.55 | 9.32 | 5.64 | 2.34 | 3.7 | 1.14 | 0.171 | 73.93 | 49.2 | N | N | Y |
| 082 | 0 | 32.97 | 5.95 | 26.22 | 0 | 3.31 | N | 20 | 7.69 | 4.974 | 1.7 | 4.33 | 1.01 | 0.157 | 59.66 | 30.8 | N | Y | N |
| 083 | 0 | 40.33 | 5.56 | 29.18 | 1 | 0.94 | Y | 12.49 | 9.96 | 5.191 | 2.3 | 4.45 | 0.94 | 0.101 | 75.84 | 31.1 | N | Y | N |
| 084 | 0 | 27.21 | 8.43 | 20.14 | 1 | 3.43 | Y | 33.38 | 13.49 | 5.556 | 3.33 | 3.84 | 1.37 | 0.138 | 78.01 | 40.3 | N | N | Y |
| 085 | 0 | 25.31 | 7.13 | 30.93 | 1 | 3.07 | N | 22.3 | 8.98 | 4.94 | 1.97 | 4.63 | 0.94 | 0.14 | 77.53 | 38.1 | N | Y | Y |
| 086 | 0 | 27.32 | 8.86 | 17.85 | 0 | 4.48 | Y | 14.62 | 9.13 | 5.637 | 2.29 | 4.89 | 1.03 | 0.094 | 82.44 | 45.1 | N | N | Y |
| 087 | 0 | 36.63 | 8.42 | 23.61 | 1 | 4.78 | Y | 23.57 | 7.6 | 5.293 | 1.79 | 4.23 | 1.08 | 0.2 | 80.11 | 37.5 | N | N | Y |
| 088 | 0 | 32.78 | 7.41 | 25.98 | 0 | 7.18 | Y | 18.55 | 6.75 | 5.245 | 1.57 | 4.41 | 1.17 | 0.175 | 76.58 | 31.8 | N | Y | N |
| 089 | 0 | 27.14 | 5.97 | 25 | 1 | 5.73 | Y | 50.5 | 10.38 | 5.636 | 2.6 | 4.3 | 0.85 | 0.131 | 71.59 | 37.5 | N | N | Y |
| 090 | 0 | 33.99 | 4.74 | 22.11 | 1 | 3.35 | Y | 41.2 | 6.74 | 5.54 | 1.66 | 3.52 | 1.39 | 0.141 | 56.59 | 40.7 | N | N | Y |
| 091 | 0 | 31.26 | 6.71 | 23.43 | 0 | 2.85 | N | 30.94 | 8.74 | 5.25 | 2.04 | 3.98 | 1.09 | 0.148 | 76.3 | 38.9 | N | N | Y |
| 092 | 0 | 36.98 | 9.12 | 22.52 | 1 | 5.02 | N | 48.11 | 7.56 | 5.797 | 1.95 | 4.33 | 1.51 | 0.058 | 74.21 | 37 | N | N | Y |
| 093 | 0 | 26.01 | 7.94 | 22.21 | 1 | 2.19 | Y | 31.92 | 9.62 | 4.991 | 2.13 | 4.26 | 1 | 0.13 | 74.81 | 40.4 | N | N | Y |
| 094 | 0 | 28.47 | 5.24 | 26.82 | 0 | 7.11 | N | 21.09 | 9.31 | 5.602 | 2.32 | 3.99 | 1.09 | 0.113 | 60.21 | 41 | N | Y | Y |
| 095 | 0 | 28.04 | 7.86 | 25.24 | 1 | 6.09 | N | 48.61 | 10.83 | 5.594 | 2.69 | 3.84 | 1.11 | 0.1 | 62.06 | 42.3 | N | N | Y |
| 096 | 0 | 21 | 8.25 | 21.88 | 1 | 3.52 | Y | 37.25 | 7.49 | 5.269 | 1.75 | 4.18 | 1.51 | 0.129 | 82.18 | 37.9 | N | N | Y |
| 097 | 0 | 32.57 | 5.9 | 26.98 | 1 | 1.58 | Y | 19.06 | 8.01 | 5.497 | 1.96 | 4.17 | 0.62 | 0.212 | 71.88 | 33 | N | Y | N |
| 098 | 0 | 32.34 | 7.82 | 25.08 | 0 | 6.36 | N | 28.83 | 6.48 | 4.93 | 1.42 | 5.27 | 1.34 | 0.166 | 78.68 | 34.9 | N | N | N |
| 099 | 0 | 30.65 | 7.65 | 26.7 | 1 | 4.07 | Y | 20.93 | 7.63 | 5.713 | 1.94 | 5.03 | 0.81 | 0.117 | 72.52 | 41.2 | N | Y | Y |
| 100 | 0 | 29.08 | 5.43 | 26.11 | 1 | 6.18 | Y | 15.07 | 9.4 | 5.313 | 2.22 | 4.11 | 1.27 | 0.163 | 72.53 | 29.1 | N | Y | N |
| 101 | 1 | 39.92 | 6.21 | 29.29 | 1 | 2.62 | N | 60.29 | 12.94 | 5.369 | 3.09 | 4.74 | 1.29 | 0.165 | 66.72 | 38.7 | Y | Y | N |
| 102 | 1 | 36.73 | 8.19 | 16 | 1 | 0.87 | Y | 53.46 | 14.35 | 6.614 | 4.22 | 3.51 | 0.69 | 0.235 | 61.28 | 49.8 | Y | N | Y |
| 103 | 1 | 30.93 | 8.38 | 33.11 | 1 | 3.16 | Y | 60.26 | 6.41 | 5.739 | 1.63 | 5.18 | 1.71 | 0.162 | 81.48 | 54.3 | Y | Y | Y |
| 104 | 1 | 26.19 | 7.93 | 25.31 | 1 | 2.12 | N | 65.45 | 13.06 | 5.303 | 3.08 | 4.45 | 1.62 | 0.28 | 83.35 | 51 | N | N | Y |
| 105 | 1 | 35.22 | 6.35 | 29.68 | 1 | 6.6 | Y | 59.35 | 15.46 | 6.272 | 4.31 | 4.04 | 0.96 | 0.178 | 71.84 | 53 | Y | Y | Y |
| 106 | 1 | 33.17 | 6.12 | 22.16 | 1 | 5.64 | N | 59.25 | 8.61 | 5.196 | 1.99 | 4 | 0.95 | 0.062 | 54.48 | 40.7 | Y | N | N |
| 107 | 1 | 36.54 | 7.37 | 27.94 | 0 | 3.08 | Y | 50.09 | 16.71 | 5.891 | 4.38 | 4.83 | 0.94 | 0.188 | 72.1 | 36 | N | Y | N |
| 108 | 1 | 34.79 | 9.7 | 22.11 | 1 | 7.52 | N | 50.58 | 13.19 | 5.863 | 3.44 | 5.51 | 1.34 | 0.169 | 80.19 | 56.5 | N | N | Y |
| 109 | 1 | 37.57 | 8.47 | 23.62 | 1 | 5.6 | N | 41.46 | 10.03 | 6.684 | 2.98 | 4.99 | 1.35 | 0.214 | 64.06 | 43.7 | Y | N | N |
| 110 | 1 | 26.94 | 6.7 | 32.5 | 1 | 3.63 | Y | 80.32 | 11.85 | 6.226 | 3.28 | 5.05 | 0.94 | 0.184 | 78.39 | 44.2 | N | Y | N |
| 111 | 1 | 39.37 | 8.92 | 25.13 | 1 | 0.5 | Y | 43.41 | 13.58 | 5.851 | 3.53 | 5.15 | 1.24 | 0.143 | 76.68 | 55.3 | N | N | Y |
| 112 | 1 | 31.86 | 4 | 26.56 | 0 | 7.58 | Y | 50.21 | 12.22 | 6.483 | 3.52 | 5.04 | 0.77 | 0.089 | 81.8 | 49.9 | Y | Y | Y |
| 113 | 1 | 44.46 | 9.65 | 28.85 | 1 | 2.02 | Y | 49.89 | 13.52 | 6.913 | 4.15 | 4.58 | 1.24 | 0.14 | 77.84 | 49.2 | N | Y | Y |
| 114 | 1 | 25.91 | 4.04 | 27.53 | 1 | 1.64 | Y | 69.78 | 9.42 | 6.288 | 2.63 | 4.22 | 1.29 | 0.107 | 68.42 | 61.2 | Y | Y | Y |
| 115 | 1 | 42.18 | 6.72 | 26.33 | 1 | 5.66 | Y | 55.11 | 12.49 | 5.878 | 3.26 | 5.19 | 0.99 | 0.121 | 87.12 | 41.5 | Y | Y | N |
| 116 | 1 | 31.86 | 5.25 | 27.08 | 1 | 7.8 | N | 74.13 | 8.04 | 4.886 | 1.75 | 4.93 | 0.86 | 0.179 | 75.44 | 52.5 | Y | Y | Y |
| 117 | 1 | 26.16 | 4.91 | 34.21 | 0 | 6.21 | N | 50.45 | 11.7 | 5.071 | 2.64 | 5.45 | 1.44 | 0.233 | 76.57 | 46.4 | N | Y | Y |
| 118 | 1 | 27.28 | 9.67 | 25.6 | 1 | 4.97 | N | 62.19 | 9.47 | 5.641 | 2.37 | 5.28 | 1.4 | 0.17 | 68.77 | 47.9 | N | N | Y |
| 119 | 1 | 28.38 | 6.51 | 26.5 | 1 | 5.02 | N | 48.45 | 9.94 | 5.413 | 2.39 | 4.68 | 1.44 | 0.09 | 72.99 | 56.7 | N | Y | Y |
| 120 | 1 | 33.38 | 8.09 | 29.48 | 1 | 5.41 | Y | 85.2 | 15.48 | 5.73 | 3.94 | 4.44 | 0.71 | 0.172 | 69.18 | 49.6 | Y | Y | Y |
| 121 | 1 | 34.04 | 7.33 | 29.67 | 1 | 5.27 | Y | 44.11 | 15.08 | 5.912 | 3.96 | 4.7 | 0.97 | 0.21 | 75.32 | 49.8 | N | Y | Y |
| 122 | 1 | 26.68 | 8.35 | 29.95 | 0 | 4.54 | N | 62.57 | 13.72 | 6.267 | 3.82 | 4 | 1.39 | 0.12 | 91.56 | 53.5 | Y | Y | Y |
| 123 | 1 | 30.37 | 7.57 | 28.04 | 1 | 4.28 | N | 66.56 | 8.94 | 5.817 | 2.31 | 4.37 | 1.51 | 0.143 | 64.98 | 43.4 | Y | Y | N |
| 124 | 1 | 44.9 | 4.93 | 21.8 | 1 | 4.03 | N | 63.85 | 11.12 | 6.235 | 3.08 | 4.14 | 1.06 | 0.151 | 62.9 | 40.1 | Y | N | N |
| 125 | 1 | 42.12 | 9.35 | 31.52 | 1 | 2.98 | N | 56.47 | 15.42 | 5.918 | 4.06 | 5.02 | 1.28 | 0.111 | 89.49 | 38.7 | Y | Y | N |
| 126 | 1 | 33.46 | 6.46 | 21.79 | 1 | 3.7 | Y | 57.15 | 12.41 | 4.388 | 2.42 | 3.84 | 1.28 | 0.182 | 75.01 | 32.2 | N | N | N |
| 127 | 1 | 30.79 | 4.4 | 26.86 | 1 | 1.56 | Y | 59.29 | 18.53 | 5.165 | 4.25 | 5.17 | 1.06 | 0.144 | 71.04 | 60.4 | N | Y | Y |
| 128 | 1 | 31.34 | 6.59 | 23.16 | 1 | 3.97 | Y | 56.94 | 13.12 | 5.762 | 3.36 | 4.77 | 0.95 | 0.171 | 85.77 | 38.3 | Y | N | N |
| 129 | 1 | 34.65 | 10.33 | 25.58 | 1 | 2.63 | N | 70.63 | 12.55 | 4.963 | 2.77 | 4.44 | 1.53 | 0.106 | 71.59 | 44.5 | Y | N | Y |
| 130 | 1 | 23.67 | 9.07 | 26.95 | 1 | 2.45 | N | 86.01 | 11.14 | 6.32 | 3.13 | 4.6 | 1.59 | 0.128 | 92.55 | 44.1 | N | Y | N |
| 131 | 1 | 28.8 | 6.74 | 26.92 | 1 | 4.39 | N | 51.88 | 12.39 | 7.018 | 3.86 | 4.69 | 1.87 | 0.186 | 82.65 | 48.2 | Y | Y | Y |
| 132 | 1 | 19.33 | 6.74 | 27.28 | 1 | 6.22 | Y | 41.86 | 11.58 | 6.784 | 3.49 | 3.8 | 1.13 | 0.131 | 72.6 | 51.7 | N | Y | Y |
| 133 | 1 | 33.21 | 6.8 | 31.45 | 1 | 5.51 | Y | 57.66 | 14 | 5.71 | 3.55 | 4.01 | 0.9 | 0.236 | 95.41 | 52 | N | Y | Y |
| 134 | 1 | 34.87 | 11.45 | 27.39 | 1 | 1.75 | Y | 90.56 | 14.7 | 6.988 | 4.57 | 4.05 | 1.25 | 0.13 | 71.59 | 41.9 | Y | Y | N |
| 135 | 1 | 27.29 | 8.16 | 24.95 | 1 | 2.63 | Y | 48.84 | 9.8 | 5.982 | 2.61 | 4.6 | 1.33 | 0.161 | 83.21 | 44.8 | Y | N | Y |
| 136 | 1 | 41.09 | 8.25 | 29.17 | 0 | 5.54 | Y | 78.96 | 8.27 | 5.617 | 2.06 | 3.99 | 1.1 | 0.197 | 79.39 | 48 | Y | Y | Y |
| 137 | 1 | 22.31 | 9.42 | 29.96 | 0 | 2.64 | Y | 79.44 | 6.85 | 5.41 | 1.65 | 4.07 | 1.48 | 0.079 | 88.03 | 44.3 | Y | Y | Y |
| 138 | 1 | 20.7 | 7.88 | 21.5 | 0 | 3.78 | Y | 51.92 | 13.44 | 5.643 | 3.37 | 4.11 | 1.14 | 0.062 | 90.27 | 57.3 | Y | N | Y |
| 139 | 1 | 32.04 | 6.92 | 27.89 | 1 | 4.95 | N | 65.77 | 8.91 | 5.579 | 2.21 | 4.75 | 1.02 | 0.074 | 77.09 | 49.4 | N | Y | Y |
| 140 | 1 | 37.56 | 7.04 | 28.25 | 1 | 2.66 | Y | 78.83 | 5.96 | 5.998 | 1.59 | 4.81 | 1.68 | 0.213 | 70.32 | 51.1 | Y | Y | Y |
| 141 | 1 | 41.83 | 7.28 | 24.76 | 1 | 4.28 | Y | 54.08 | 13.1 | 5.99 | 3.49 | 4.59 | 1.47 | 0.122 | 80.77 | 50.6 | N | N | Y |
| 142 | 1 | 27.45 | 7.35 | 30.61 | 1 | 2.11 | Y | 56.24 | 19.02 | 5.872 | 4.96 | 3.57 | 1.07 | 0.278 | 70.97 | 54.2 | N | Y | Y |
| 143 | 1 | 37.77 | 8.83 | 25.27 | 1 | 3.21 | Y | 51.58 | 11.99 | 6.198 | 3.3 | 4.58 | 0.88 | 0.122 | 94.17 | 47.3 | N | N | Y |
| 144 | 1 | 30.27 | 7.52 | 26.24 | 1 | 4.6 | Y | 46.24 | 14.37 | 6.763 | 4.32 | 4.59 | 1.52 | 0.159 | 77.99 | 36.9 | N | Y | N |
| 145 | 1 | 29.37 | 8.84 | 25.19 | 1 | 1.71 | Y | 50.74 | 12.24 | 6.582 | 3.58 | 3.65 | 0.84 | 0.227 | 73.66 | 48.8 | N | N | Y |
| 146 | 1 | 36.46 | 7.26 | 25.85 | 1 | 4.79 | Y | 43.66 | 11.73 | 4.756 | 2.48 | 4.58 | 1.34 | 0.25 | 78.4 | 41.4 | Y | N | N |
| 147 | 1 | 34.91 | 7.57 | 21.96 | 1 | 4.09 | Y | 59.17 | 14.59 | 3.955 | 2.56 | 4.83 | 1.23 | 0.253 | 65.2 | 42.3 | Y | N | N |
| 148 | 1 | 19.95 | 4 | 20.76 | 1 | 4.95 | Y | 49.26 | 11.19 | 6.571 | 3.27 | 4.71 | 1.47 | 0.21 | 68.43 | 50.1 | Y | N | Y |
| 149 | 1 | 40.44 | 9.2 | 31.38 | 1 | 4.46 | Y | 61.63 | 12.76 | 4.845 | 2.75 | 4.31 | 1.02 | 0.201 | 87.16 | 54.7 | N | Y | Y |
| 150 | 1 | 39.79 | 8.09 | 22.84 | 1 | 6.18 | N | 39.57 | 12.91 | 5.701 | 3.27 | 4.42 | 1.26 | 0.18 | 71.15 | 46.1 | Y | N | Y |

**Abbreviations:** BMI – body mass index; PCOS – polycystic ovary syndrome; HOMA-IR – homeostasis model assessment of insulin resistance; SAS – Self-Rating Anxiety Scale; FINS – fasting insulin; FPG – fasting plasma glucose.

**Note:** Data are de-identified; all patient identifiers have been removed. Continuous variables are presented as raw observed values at enrollment. “Outcome” indicates pregnancy result within 12 weeks after threatened abortion diagnosis.
